# Russell bodies in a skin biopsy: a case report

**DOI:** 10.1186/1752-1947-3-108

**Published:** 2009-11-04

**Authors:** Joanne Verheij, Elisabeth H Jaspars, Paul van der Valk, Lawrence Rozendaal

**Affiliations:** 1 Department of Pathology, VU University Medical Center, De Boelelaan 1117, 1081 HV Amsterdam, The Netherlands

## Abstract

**Introduction:**

The presence of eosinophilic bodies in a skin biopsy can be found in a variety of situations and this may present a challenge to the pathologist. The differential diagnosis of these eosinophilic structures include microorganisms such as histoplasmosis or cryptococcosis, fungi, Michaelis-Gutmann bodies, deposits of amyloid or immunoglobulins, colloid bodies or elastic bodies.

**Case presentation:**

During a routine examination of a skin biopsy with actinic keratosis taken from the cheek of a 61-year-old man, clusters of eosinophilic bodies were seen within an inflammatory infiltrate in the dermis, both intracytoplasmic and extracellular. Using additional immunohistochemical staining, these structures were identified as polyclonal Russell bodies.

**Conclusion:**

The differential diagnosis of intracytoplasmic eosinophilic structures in a skin biopsy includes Russell bodies, an uncommon finding that may be associated with chronic inflammatory conditions.

## Introduction

Russell bodies are considered to be aggregated unreleased immunoglobulin components, as a result of a block in the normal secretion pathway of immunoglobulins. They are stored within the rough endoplasmic reticulum of plasma cells and may totally fill up the cytoplasm and compress the nuclei. They mainly accumulate in plasma cells but may exist as smaller particles in extracellular locations as well [1,2]. Intracytoplasmic Russell bodies or intranuclear Dutcher bodies may be seen either in the presence of a lymphoplasmacellular inflammatory process with a polyclonal immunoreactive pattern of the plasma cells, or in the context of plasmacytoma or multiple myeloma. A wide variety of inflammatory or even neoplastic skin disorders can be accompanied by a rather extensive lymphoplasmacellular infiltrate (for example, syphilis, syringocystadenoma papilliferum and rhinoscleroma), but Russell bodies are not a conspicuous feature, with the possible exception of rhinoscleroma [3]. The differential diagnosis of Russell bodies includes organisms such as histoplasmosis and cryptococcosis, fungal organisms and Michaelis-Gutmann bodies found in histiocytes in the setting of malakaplakia. Other eosinophilic structures in the skin include amyloid deposits, colloid bodies resulting from degenerated keratinocytes and often associated with a lichenoid reaction pattern, and PAS-positive elastic bodies that can be found in the setting of lupus erythematosus or scleroderma [1,3].

## Case presentation

**Figure 1:**
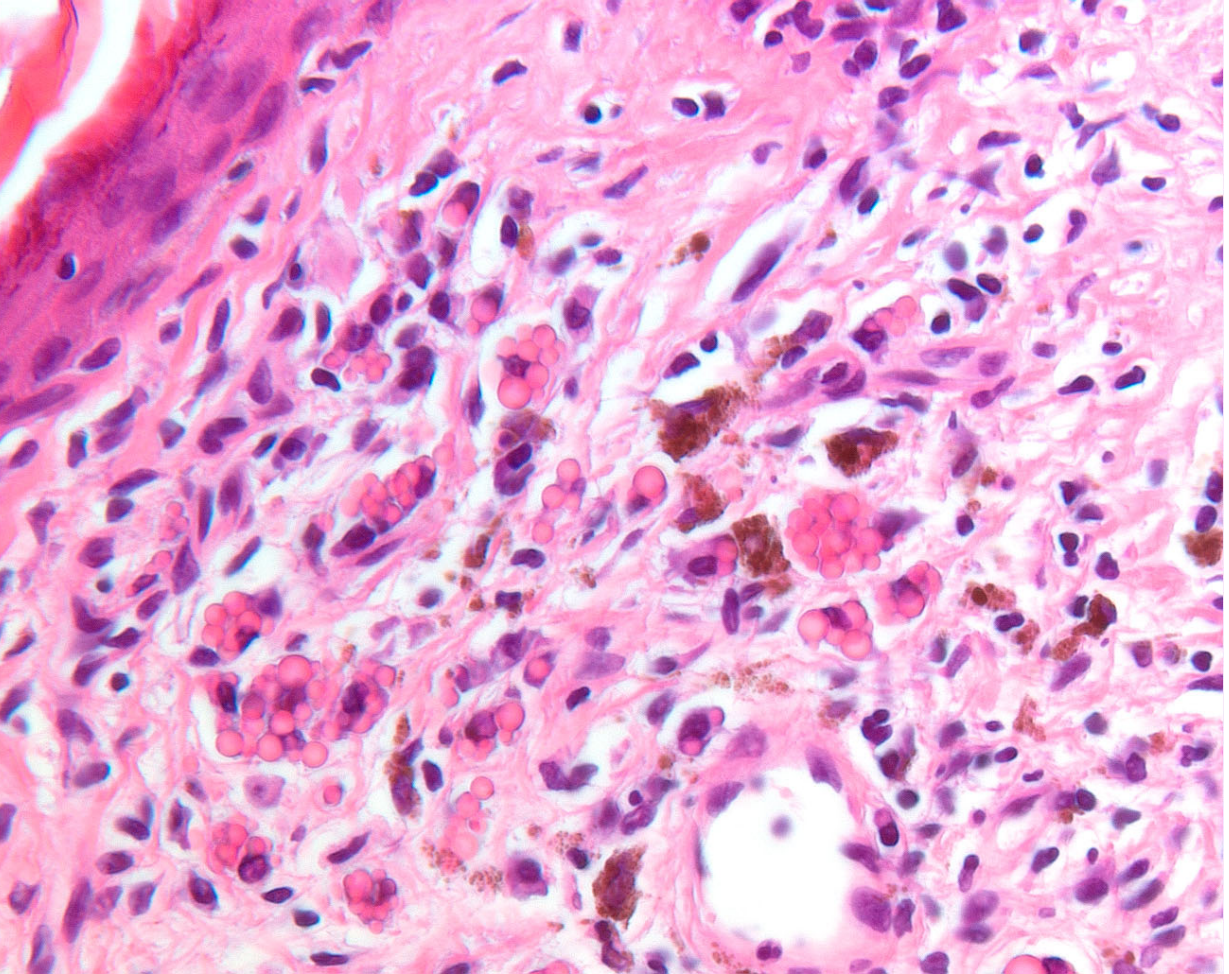
**Clusters of plasma cells with intracytoplasmic Russell bodies and independent eosinophilic structures in the dermis**.

**Figure 2:**
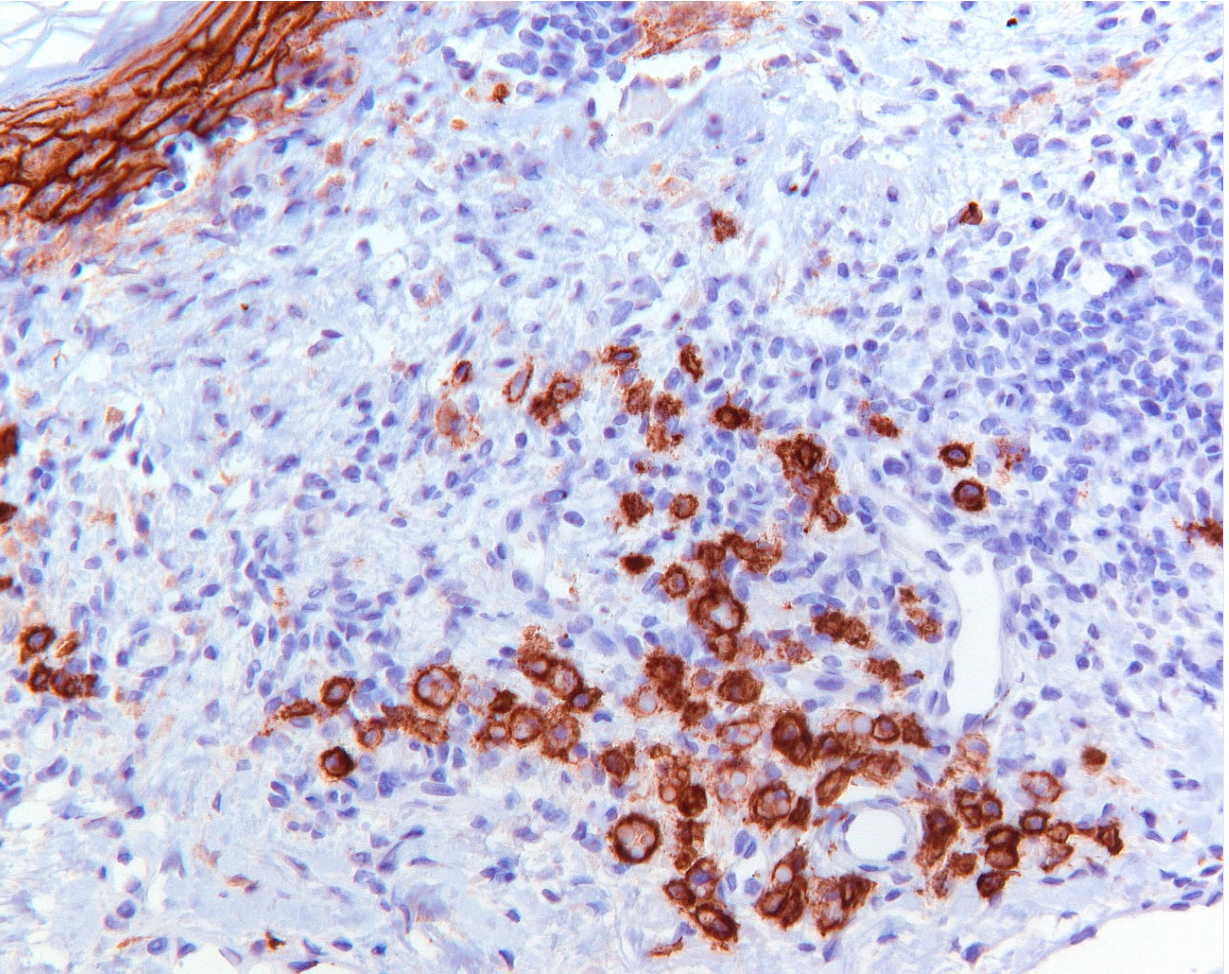
**Positive immunohistochemical staining of the plasma cells with CD138**.

A biopsy was taken from a hyperkeratotic lesion on the cheek of a 61-year-old man and submitted for routine histological examination with a differential diagnosis of actinic keratosis or basal cell carcinoma. In the first slide, a chronic inflammation was seen in the upper dermis in the presence of melanophages. Serial slides were ordered in which round homogeneous eosinophilic structures were present in the dermis (Figure 1). These bodies varied in size measuring a median of 4.2 μm (range 1.6-10.3 μm). We performed a CD138, kappa and lambda staining. Most of the structures appeared to be present within the cytoplasm of plasma cells, so-called Mott cells, as shown by CD138 staining (Figure 2). The eosinophilic structures were identified as Russell bodies. The plasma cells had a polyclonal immunoreactive pattern with positive staining for either lambda or kappa. In the serial slides, we also found the primary lesion, which was an actinic keratosis, with dysmaturation of the basal and supra-basal (spinous) layers, hyperkeratosis, parakeratosis and elastotic changes in the dermis.

## Discussion

Actinic keratosis is known to be associated with a moderate to prominent lymphoplasmacellular infiltrate [4,5]. The presence of Russell bodies in a skin biopsy in an inflammatory setting is quite rare and was described 20 years ago [1]. However, in that case, the majority of the bodies were present as independent structures in the neighbourhood of partly damaged plasma cells, whereas in our biopsy, the bodies appeared to be present both within the cytoplasm of intact plasma cells (CD138 staining) and as independent structures. This may explain the larger variability in size of the structures found in our biopsy as compared with independent so-called plasma cell bodies that measured up to 5 μm [1]. *Helicobacter pylori *gastritis, being a chronic inflammatory condition, has also been associated with the presence of Russell bodies [6-9]. The differential diagnosis of Russell bodies includes organisms such as histoplasmosis and cryptococcosis, fungal organisms and Michaelis-Gutmann bodies found in histiocytes in the setting of malakaplakia. These diagnoses were excluded in our skin biopsy using a periodic acid-Schiff, Grocott and CD68 stain, respectively.

In our patient, the primary cause of the lymphoplasmacellular infiltration was found to be an actinic keratosis. Syphilis, as possible cause for the presence of plasma cells, was excluded using additional immunohistochemical stains.

## Conclusion

The differential diagnosis of intracytoplasmic eosinophilic structures in a skin biopsy includes Russell bodies, an uncommon finding that may be associated with chronic inflammatory conditions.

## Consent

Written informed consent was obtained from the patient for publication of this case report and any accompanying images. A copy of the written consent is available for review by the Editor-in-Chief of this journal.

## Competing interests

The authors declare that they have no competing interests.

## Authors' contributions

JV, EHJ, PvdV and LR all participated in interpretation, intellectual content and drafting of the manuscript. All authors have read and approved the manuscript.
